# Spatial transcriptomics: a new frontier in accurate localization of breast cancer diagnosis and treatment

**DOI:** 10.3389/fimmu.2024.1483595

**Published:** 2024-10-08

**Authors:** Yang Zhang, Shuhua Gong, Xiaofei Liu

**Affiliations:** ^1^ Breast and Thyroid Surgery, Shandong University of Traditional Chinese Medicine Affiliated Hospital, Jinan, China; ^2^ Department of Student Affair, Shandong College of Traditional Chinese Medicine, Yantai, China

**Keywords:** breast cancer, spatial transcriptomics, tumor heterogeneity, tumor microenvironment, immunotherapy

## Abstract

Breast cancer is one of the most prevalent cancers in women globally. Its treatment and prognosis are significantly influenced by the tumor microenvironment and tumor heterogeneity. Precision therapy enhances treatment efficacy, reduces unwanted side effects, and maximizes patients’ survival duration while improving their quality of life. Spatial transcriptomics is of significant importance for the precise treatment of breast cancer, playing a critical role in revealing the internal structural differences of tumors and the composition of the tumor microenvironment. It offers a novel perspective in studying the spatial structure and cell interactions within tumors, facilitating more effective personalized treatments for breast cancer. This article will summarize the latest findings in the diagnosis and treatment of breast cancer from the perspective of spatial transcriptomics, focusing on the revelation of the tumor microenvironment, identification of new therapeutic targets, enhancement of disease diagnostic accuracy, comprehension of tumor progression and metastasis, assessment of drug responses, creation of high-resolution maps of tumor cells, representation of tumor heterogeneity, and support for clinical decision-making, particularly in elucidating the tumor microenvironment, tumor heterogeneity, immunotherapy and their correlation with clinical outcomes.

## 1 Introduction

**Figure 1:**
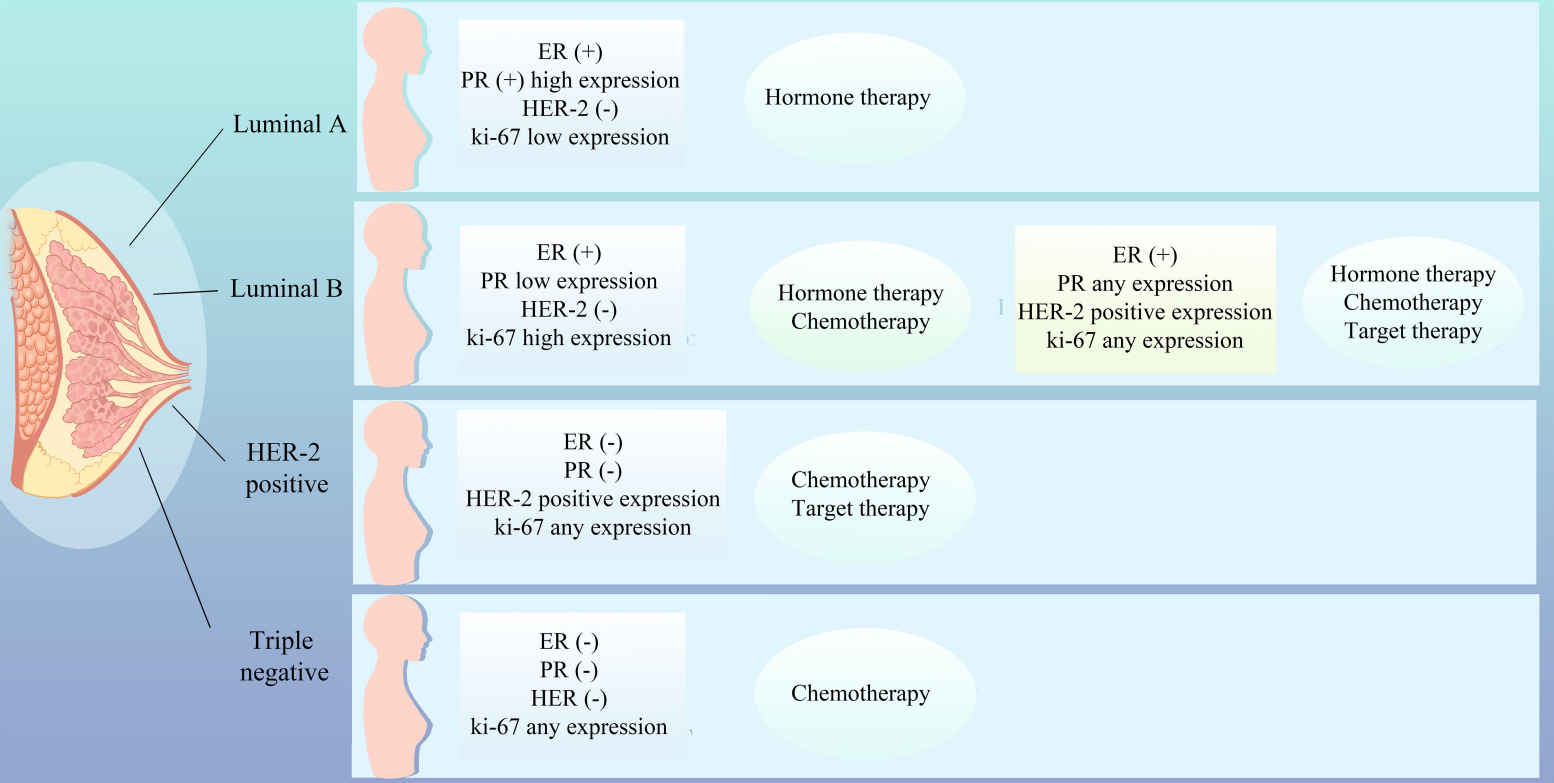
Molecular subtyping of breast cancer and therapeutic strategies.

The therapeutic approach to breast cancer necessitates a personalized strategy that accounts for the patient’s unique profile, encompassing the tumor’s biological attributes, genomic expression signatures, estrogen and progesterone receptor status, HER2 amplification, tumor microenvironmental dynamics, the patient’s genetic predispositions, and lifestyle factors (1). Breast cancer is stratified into distinct molecular subtypes, including Luminal A, Luminal B, HER2-positive, and triple-negative breast cancer (TNBC), each exhibiting divergent responses to therapeutic interventions, thereby underscoring the imperative for tailored treatment modalities such as chemotherapy, hormonal therapies, targeted therapies, and immunotherapies (**Figure 1**). Molecular subtyping also serves as a prognostic indicator, providing insights into the survival outcomes of breast cancer patients (2). Accurate molecular subtyping is pivotal for the formulation of effective treatment regimens. However, the intrinsic heterogeneity of breast cancer, characterized by variable gene expression and mutational landscapes among patients with ostensibly similar disease, may lead to therapeutic resistance, disease progression, prognostic variability, and clonal evolution, thereby advocating for individualized therapeutic approaches (3).

Precision oncology in breast cancer is witnessing a transformative evolution, propelled by the remarkable advancements in genomics, biomarker identification, and tailored therapeutic approaches. The meticulous genomic profiling of neoplastic tissues has facilitated the elucidation of pivotal molecular targets, thereby enabling the formulation of more precise and efficacious treatment regimens for affected individuals. Notably, immunotherapeutic interventions, particularly the utilization of immune checkpoint inhibitors (4), have emerged as a potent therapeutic modality for select breast cancer subtypes. Furthermore, the application of mitochondrial inhibitors (5) has demonstrated the potential to enhance the therapeutic efficacy by modulating the metabolic reprogramming of breast cancer cells. The incorporation of artificial intelligence (AI) and machine learning (ML) algorithms has revolutionized the analysis of extensive datasets, thereby unveiling novel predictive models that forecast treatment responsiveness and disease progression (6–8). Concurrently, the integration of lifestyle modifications and pharmacological interventions as preventive strategies (9) underscores the multifaceted and individualized nature of breast cancer management. Collectively, these developments herald an era of expanded therapeutic horizons and renewed optimism for breast cancer patients.

Advancements in oncology research have been significantly propelled by the advent of Spatial Transcriptomics (ST), a cutting-edge technology that enables the concurrent assessment of gene expression profiles in the context of cellular spatial architecture (10). ST has emerged as a valuable tool for dissecting tumor heterogeneity, thereby facilitating a more precise understanding of tumor progression and therapeutic responses. Its utility extends to the elucidation of tumor microenvironment (TME) intricacies, offering insights into the spatial distribution and reciprocal interactions between neoplastic and surrounding cells (11). ST has been instrumental in revealing the complex interplay between tumor cells and the immune milieu, as well as in delineating the gene expression patterns within the immune microenvironment, thereby enhancing our comprehension of immune function. For instance, ST has delineated five distinct immune microenvironmental subtypes in hepatocellular carcinoma, uncovering the heterogeneity of tumor-associated neutrophils (TAN) and identifying key subpopulations, such as CCL4^+^ and PD-L1^+^ TAN, which are implicated in tumor promotion, thus suggesting novel therapeutic targets for liver cancer (12). Utilizing ST, Ye et al. have delineated the tumor boundary region, which serves as a critical interface between malignant and non-malignant tissues, identifying specific cell subtypes, cellular interactions, and potential therapeutic targets enriched at this boundary, and have observed significant infiltration of FAP^+^ fibroblasts and SPP1^+^ macrophages in colorectal cancer, which correlates with adverse prognosis and resistance to immunotherapy (13, 14).

In the field of breast cancer research, the application of ST is gradually demonstrating its unique value and potential. Although the current research articles exhibit a certain degree of dispersion, each contains valuable information and insights that urgently await systematic organization and integration. This review aims to synthesize these research findings into a comprehensive knowledge map through meticulous literature review and in-depth analysis, with the expectation of revealing the integrated application and development trends of ST in breast cancer research.

Furthermore, this study is dedicated to exploring the potential application prospects of ST in breast cancer research, as well as the innovative and transformative impacts it may bring. Through this process, we anticipate providing new perspectives and ideas for understanding the biological mechanisms of breast cancer, improving diagnostic methods, and optimizing treatment strategies.

## 2 ST and the molecular characteristics of breast cancer

Breast cancer is marked by pronounced tumor heterogeneity, with neoplastic cells from disparate regions potentially manifesting divergent gene expression profiles and biological behaviors. A research consortium from the Australian Institute for Medical Research has harnessed ST in conjunction with single-cell RNA sequencing to delineate the most exhaustive cellular cartography of breast cancer to date. This investigation has not only unveiled the heterogeneity of neoplastic cells but also delineated nine tumor ecotypes correlated with the overall patient survival, with certain ecotypes being predictive of an adverse prognosis (15).

Invasive micropapillary carcinoma (IMPC), a distinctive histological variant of breast cancer, is distinguished by its high propensity for lymphovascular invasion and lymph node metastasis. A team led by Lv, J. has postulated the “IMPC tumor cell clump metastasis” hypothesis, pioneering the transcriptional profiling of IMPC and uncovering its profound heterogeneity, which is intricately linked to metabolic reprogramming. Metabolically aberrant IMPC subpopulations are spatially segregated and exhibit heightened lipid metabolism across all IMPC stratified clusters. Concurrently, elevated expression of the sterol regulatory element-binding transcription factor 1 (SREBF1) protein has been correlated with increased lymph node metastasis and diminished survival rates in IMPC patients, underscoring its potential as a diagnostic and therapeutic biomarker (16).

Yoshitake, R. and colleagues have elucidated that estrogen receptor-positive (ER+) breast cancer encompasses four spatially discrete populations with functional heterogeneity, including estrogen responsiveness, proliferation, hypoxia induction, and inflammation association. The “proliferative” subset is pivotal for estrogen-driven tumorigenesis, conferring a phenotype reminiscent of the luminal B subtype. Gene signatures emanating from proliferative, hypoxia-induced, and inflammation-associated populations are significantly associated with inferior clinical outcomes, whereas patients with estrogen-responsive signatures demonstrate a more favorable prognosis (17).

Sun H (18) associates have stratified the MDA-MB-231 tumor mass into necrotic, peripheral necrotic, hypoxic tumor, adaptive survival tumor, and invasive tumor compartments based on hypoxic status and transcriptomic profiling. Each compartment possesses a unique expression signature, with diverse gene networks activated under the influence of distinct hypoxic microenvironments, thereby dictating the fate of tumor cells across different regions. The spatial transcriptional distribution of 35 hypoxia-associated genes was mapped, revealing that disparate tumor regions with distinct hypoxia-related gene signatures exhibit unique characteristics. B lymphocytoma-2 gene-homology 3 (BINP3), implicated in the regulation of apoptosis, exhibits heightened expression in hypoxic regions, whereas prolyl-4-hydroxylase alpha polypeptide I (P4HA1), though broadly expressed, displays significant variation at the periphery of necrotic areas. Lactate dehydrogenase (LDHA), a marker of tumor metabolism, is ubiquitously upregulated across the tumor tissue, with the most pronounced differences in the invasive regions. Beyond hypoxia-inducible factor-1α (HIF-1α), alternative pathways are implicated in the modulation of these gene networks. These hypoxia-associated genes not only interact among themselves but also serve as key regulators within the gene regulatory networks of each compartment. The elucidation of these spatial heterogeneities is instrumental for advancing our understanding of breast cancer biology and for the development of novel therapeutic strategies targeting breast malignancies.

The architecture and functionality of mammary tissue exhibit intricate spatial complexity, with cellular constituents and states across regions potentially exerting distinct influences on the health of the breast and the evolution of pathologies. Precise stratification is paramount for the determination of appropriate therapeutic interventions. Research has posited that the heterogeneity of cellular populations within neoplastic tissue, encompassing both malignant and non-malignant entities, are organized into tumor regions or “niches” with distinct cellular compositions. These zones may reflect the recruitment of specific cellular subpopulations or the differentiation processes of cells (3). Kumar, T.’s investigative research has elucidated the molecular disparities between the ductal and alveolar compartments of the breast, as well as the ecosystem of resident immune cells within these tissues. Specifically, certain subtypes of immune and basal cells exhibit a higher prevalence in the ductal and alveolar regions, in contrast to their sparse distribution in the connective tissue areas. The ductal region has been correlated with an enhanced expression of genes associated with secretory luminal epithelial cells (LumSec), whereas the alveolar region is characterized by an upregulation of genes specific to hormone-responsive luminal epithelial cells (LumHR). Lymphocytes were predominantly detected in the connective tissue areas, whereas vascular cells showed a higher prevalence in the ductal and alveolar regions (19).

An additional study employing single-cell RNA sequencing (scRNA-seq) and ST has identified a cluster of disseminating cancer cells characterized by heightened oxidative phosphorylation (OXPHOS) activity. This investigation has discerned a metabolic shift between glycolysis and OXPHOS as the process of dissemination commences. Moreover, this distinctive cellular cluster is observed to be distributed along the tumor’s leading edge (20). The heterogeneity of cellular positions at the tumor ductal periphery or core underscores the necessity of incorporating the spatial architecture of the tumor in therapeutic strategies. Spatially resolved transcriptomics, genomics, and single-cell analyses have unveiled the intrinsic subtype heterogeneity within mixed infiltrating ductal and lobular carcinoma (MDLC). Compared to TNBC or basal ductal and estrogen receptor-positive (ER^+^) phenotypes, MDLC exhibits a pronounced enrichment of luminal lobular region cells characterized by cell cycle arrest/senescence and oncogenic (ER and MYC) features, along with inactivation of E-cadherin 1 (CDH1) specific to the lobular rather than ductal regions. Furthermore, the identification of a unique oncogenic single-cell ductal and lobular subset accentuates the heterogeneity within the region. It has been substantiated that the tumor morphology and histological heterogeneity within MDLC are governed by intrinsic subtype and oncogenic heterogeneity, which may engender prognostic ambiguity and therapeutic challenges (21).

ST has been instrumental in elucidating the inter-regional interactions within human breast cancer tumors, as well as the regulatory mechanisms from receptor-ligand (LR) interactions to target gene expression. This approach has unveiled the intricate crosstalk between disparate cell types, which may exert substantial influence on the functional attributes of mammary tissue and the trajectory of disease progression. In a study conducted by Wang H et al., it was determined that multi-tiered signaling networks exist between any two tumor regions, with the affinity of LR interactions within these networks varying significantly between different regions (22). Certain LR pairs, such as Tumor Necrosis Factor (TNF)-Tumor Necrosis Factor Receptor Superfamily Member 21 (TNFRSF21), Retinol Binding Protein 4(RBP4)- Stimulated by Retinoic Acid Gene 6 (STRA6), Platelet-derived Growth Factor A (PDGFA)-Platelet Derived Growth Factor Receptor Beta (PDGFRB), Tenascin C (TNC)-Contactin 1 (CNTN1), and ALK and LTK ligand 2 (ALKAL2)-Anaplastic Lymphoma Kinase (ALK), have demonstrated enhanced interaction profiles. Prior research has underscored the significance of ALKAL2-ALK signaling (23, 24) and PDGFA-PDGFRB signaling (25) in the oncogenic processes of breast cancer, particularly in tumor growth and metastasis.

Employing ST analysis, investigators have discerned subpopulations of cells and molecular signatures that correlate with clinical outcomes, thereby introducing novel biomarkers pivotal for the stratification, treatment response prediction, and personalized therapeutics in breast cancer. Ductal carcinoma *in situ* (DCIS) represents an incipient phase of breast cancer with the potential to evolve into invasive ductal carcinoma. Dr. Satoi Nagasawa from the University of Tokyo performed ST analysis on DCIS, revealing that mutations in GATA Binding Protein 3 (GATA3) and Phosphoinositide-3-Kinase, Catalytic, Alpha Polypeptide (PIK3CA) are the most prevalent within this cohort. DCIS cells harboring GATA3 mutations have been observed to occasionally evolve into invasive cancers, implicating their role in epithelial-mesenchymal transition (EMT) and angiogenesis. In contrast, DCIS cells with PIK3CA mutations do not progress to malignancy (26), highlighting the cellular heterogeneity intrinsic to breast cancer tumors. ST sequencing data corroborate that DNA Damage-Inducible Transcript 3 (DDIT3) co-localizes with biomarkers of malignant epithelia (KRT19), myofibroblasts (ACTA2), and monocytic/macrophage populations (CD68), exhibiting heightened expression within cellular clusters. DDIT3’s role in modulating the TME and intercellular communication is multifaceted, with positive correlations observed with pathways implicated in apoptosis, cell cycle regulation, DNA damage response, and the epithelial-mesenchymal transition (EMT) in breast cancer (27).

## 3 ST and the breast cancer TME

TME is a complex ecosystem comprising a diverse array of immune and stromal cells, vascular structures, extracellular matrix (ECM) components, and an array of soluble mediators (28). This multifaceted milieu is instrumental in modulating tumorigenesis and dictating the trajectory of cancer evolution. The cellular constituents of the TME exhibit significant heterogeneity, and their spatial architecture varies across distinct genomic subtypes of breast cancer. These variations are manifested in the interactions and topographical arrangements among cellular subsets, which in turn can significantly influence the neoplastic process and the tumor’s responsiveness to therapeutic interventions. ST has elucidated the intricate distribution of these cellular elements within the TME and delineated the nuanced interactions between diverse cellular populations and malignant cells, thereby enhancing our comprehension of the molecular underpinnings of breast cancer and the intricacies of the TME, including its therapeutic resistance.

Investigators have meticulously mapped the spatial organization and context-specific landscape of breast cancer and its attendant microenvironment, profiling the expression of 37 proteins across a cohort of 483 tumor samples, these data helped the investigators to distinguish between different cellular phenotypes such as tumor cells, stromal cells and immune cells. For example, they were able to distinguish between different phenotypes of epithelial cells, fibroblasts, myofibroblasts, endothelial cells, T cells, B cells, and macrophages, among others, thereby unmasking the spatial heterogeneity of cellular constituents within the TME (29). Research endeavors have further characterized the spatial co-occurrence and interplay of various cellular phenotypes within breast cancer. Croizer H (30) has expounded on the malleability of FAP^+^ cancer-associated fibroblasts (CAFs) and their intricate crosstalk with immune cells, identifying a spectrum of 10 spatially orchestrated FAP^+^ CAF clusters associated with cellular modules, designated as EcoCellTypes (ECTs). These ECTs, which include immunosuppressive and immuno-permissive variants, encompass specific FAP^+^ CAF clusters and immune cell populations that are situated at discrete distances from tumor conglomerates and vascular structures. Certain FAP^+^ CAF clusters have been correlated with the invasive properties of breast cancer, suggesting that the heterogeneity among FAP^+^ CAFs may play a pivotal role in the progression of DCIS. Another study focusing on CAFs has delineated the spatial organization of disparate CAF populations within breast cancer. Some specific CAF populations were found to coincide with heightened transforming growth factor-β (TGF-β) signaling, with elastin microfibril interface-derived protein 1 (EMILIN1) emerging as a paramount regulatory gene. Elevated EMILIN1 expression at the tumor periphery is associated with robust CD8 T-cell infiltration, and such increased EMILIN1 expression correlates with an improved prognosis in breast cancer patients, underscoring its functional relevance in the recruitment of cytotoxic T cells to the TME (31).

### 3.1 ST and the tumor-immune microenvironment

The immunological components of the TME are under intense scrutiny due to their significant roles in oncological processes. Immune infiltration within tumor tissues surpasses that observed in normal tissues (32), with the potential to either combat or propagate tumorigenesis. For instance, TAN can stimulate the proliferation and invasiveness of malignant cells, yet they are also capable of exerting cytotoxic effects against tumor cells (33). Macrophages within the TME demonstrate diverse polarization states; the classically activated M1 macrophages are associated with anti-tumor activities, while the alternatively activated M2 macrophages are more inclined to support tumorigenesis (34). ST has delineated the distribution patterns of these cellular elements across tumor tissues, thereby providing a more refined representation of the spatial tumor-specificity inherent to each cell population. The composition of the TME is subject to variation predicated on spatial positioning, which corresponds to the distinct functionalities of immune cells. Research team reclustered immune cells to identify T cells and innate lymphoid cells, myeloid cells, B cells and plasmablasts. They identified 18 T-cell and innate lymphoid clusters, 13 clusters myeloid cells, three major cell types in the stromal compartment across patients, these cell clusters are mutually exclusive in their spatial arrangement, and the “ecotypes” composed of different cell clusters differ significantly from the tumor subtypes and prognosis (15). Research (19) has disclosed that immune cells are predominantly localized to the parenchymal compartments of mammary tissue, distinct from the intravascular locales, indicative of their tissue-residency. Furthermore, extensive ligand-receptor interactions between immune cells and other cellular constituents of the mammary tissue, such as epithelial and stromal fibroblasts, suggest an influential role for immune cells in the steady-state and pathological mechanisms of mammary tissue. In a cohort of 152 HER2^+^ ductal breast carcinomas, cellular constituents of the TME displayed well-defined three-dimensional localization patterns; for instance, T lymphocytes exhibit a propensity to aggregate perivascularly and along vascular networks, whereas macrophage accumulations manifest distinct distributional configurations, ranging from uniform dispersion to localized aggregation (35). Within TNBC, there exists a pronounced variability in the spatial distribution of immune cell subpopulations. Intraepithelial T and B lymphocytes consistently exhibit a more clonal and less diverse immune repertoire compared to their stromal counterparts. Overamplification of T cell clones within the intraepithelial compartment is more pronounced than within the stromal T cells, indicative of an enriched accumulation of antigen-specific T cells at the tumor core (36).

Utilizing spatial information, we can more adeptly investigate the intricate interplay between immune cells and neoplastic cells. By amalgamating single-nucleus RNA sequencing (snRNA-seq) with ST datasets to elucidate the spatial heterogeneity of immune cells within the breast cancer TME, the ST datasets have been stratified into six principal zones: the luminal region, basal region, the interfacing area between the luminal region and basal region, stroma and infiltrating lymphocyte areas based on the principal component scores across all ST spots (37). Neutrophils were found to be enriched in the luminal region, whereas B cells were observed to be primarily infiltrating the basal region. Activated CD8^+^ T cells display an enhanced tumor spatial specificity relative to their quiescent counterparts, aligning with their purported anti-tumor capabilities. Certain immunosuppressive cell populations, such as Regulatory T cells (Tregs) and Cancer-associated Fibroblasts (CAFs), exhibit diminished tumor spatial specificity. Furthermore, macrophage clusters expressing both M1 and M2 phenotypic markers, notably Mac.FABP5^+^ cells, demonstrate heightened tumor spatial specificity in comparison to pro-inflammatory macrophages, implying a potential direct induction by neoplastic cells (32). Among HER2 positive patient cohorts, a shared spatial expression signature has been identified, with reciprocal interactions between Mø and T cell subsets evident within the context of type I interferon responses (38). Collectively, these insights underscore the qualitative disparities among immune cell clusters across distinct clinical subtypes, augmenting our comprehension of the intricate interactions between tumor cells and the TME’s architectural intricacies, thereby highlighting the imperative for subtype-specific targeted therapeutic strategies in clinical practice.

### 3.2 ST and the tumor immune evasion

Recent research in the realm of tumor immune evasion has made significant strides. It is now understood that the intricate interplay among immune cells within TME, encompassing T lymphocytes, macrophages, and regulatory Treg, is crucially linked to the occurrence of immune evasion. Treg are known for their role in curbing the activity of immune effector cells, thus preventing unwarranted tissue damage and quelling inflammatory responses. However, within the inflammatory milieu of a tumor, Treg can undergo reprogramming that augments their suppressive capabilities, leading to a state that either facilitates tumor immune evasion or fosters tumor progression. Strategies that aim to diminish the Treg cell population or attenuate their activity within the tumor’s inflammatory TME, while simultaneously impeding their reprogramming, have been shown to bolster the body’s anti-tumor immune response (39). Notably, the identification of novel PD-L1^+^/PD-L2^+^ macrophage populations that correlate with clinical outcomes suggests that these macrophages might modulate immune responses in the TME through interactions with the T cell surface, playing a significant role in tumor progression and immune evasion (15). Furthermore, in basal-like tumors, epithelial cells under hypoxic conditions have been linked to the upregulation of CD274 and the downregulation of B2M, establishing a connection between hypoxia and the mechanisms underlying immune evasion (29). These findings underscore the complexity of the TME and highlight potential targets for therapeutic intervention to counteract tumor immune evasion.

ST technology facilitates a sophisticated delineation of the genotype and phenotype of diverse immune cell populations and their states of activation or suppression, unveiling novel therapeutic targets for breast cancer intervention. Claudin-low breast cancer is characterized by a pronounced immune cell infiltration, with heightened presence of B cells, T cells, NK cells, macrophages, and neutrophils relative to other breast cancer subtypes. However, clinical investigations have demonstrated that despite abundant lymphocytic infiltration, a significant number of patients exhibit resistance to immune checkpoint therapies. Beyond CD274, a repertoire of additional immune checkpoint genes, including CD276 and Neuropilin-1 (NPR1), contribute to immunosuppressive mechanisms, thereby circumscribing the efficacy of PD-L1 inhibitory agents (40). In the context of metaplastic breast cancer (MBC), there is evidence of intratumoral permeation by Treg cells, M2-macrophages, and myeloid-derived suppressor cells (MDSCs), which orchestrate an immunosuppressive milieu replete with EMT and hypoxic elements. The interplay with Treg cells is shown to be mediated through signaling pathways involving fibroblast growth factor 2 (FGF2), fibroblast growth factor receptor 1 (FGFR1), and CD44, underscoring the potential therapeutic efficacy of interventions directed at Treg cells in MBC (41). Li CJ et al. (42), employing ST technology, identified an elevated expression of the mitochondrial calcium uniporter (MCU) within tumorigenic regions. Subsequent analyses revealed a positive correlation between MCU expression and the upregulation of pivotal T cell regulatory factors. Within the BRCA invasion cohort, a significant positive association was observed between T cell infiltration and MCU expression, suggesting that MCU not only offers prognostic insights into disease progression but also serves as an indicator of immune status.

## 4 ST in relation to breast cancer treatment response and guidance of clinical strategies

ST has elucidated subpopulations of cells correlated with tumor metastasis and chemotherapy resistance, enhancing our understanding of the molecular underpinnings of these intricate biological processes. By examining the TME-modulated pharmacological responses, it is possible to predict which patients are poised to garner clinical benefit from immunotherapeutic interventions. Employing single-cell transcriptomics in conjunction with spatial proteomics, the therapeutic efficacy of pembrolizumab in TNBC has been assessed. Tumors that were refractory to treatment demonstrated a dearth of immune cell infiltration both prior to and subsequent to therapy, alongside minimal alterations in immune profiles induced by treatment. In contrast, tumors that responded to therapy could be segregated into two distinct cohorts based on pre-treatment characteristics; one cohort was characterized by elevated expression of major histocompatibility complex molecules and the presence of tertiary lymphoid structures, indicative of pre-existing anti-tumor immune activity. The other cohort, akin to non-responders at baseline, exhibited a pronounced immune response following combined therapeutic intervention, marked by the interactive engagement of cytotoxic T lymphocytes and antigen-presenting myeloid cells (43). Following neoadjuvant therapy in patients with HER2-positive tumors, significant changes occur within the immunological landscape of the tumor. These changes are characterized by a substantial decrease in HER2 expression and its downstream Akt signaling, along with an increased expression of CD45 and CD8, which corresponds to the infiltration of leukocytes and cytotoxic T cells, respectively. Conversely, cases that did not achieve pathological complete response (pCR) are marked by an increase in CD56 expression, which may suggest the lysis of chemotherapy-stressed tumor cells by natural killer (NK) cells (44). ST has been harnessed to investigate the contribution of TNBC tumor cells to the response to neoadjuvant chemotherapy (45), revealing in pCR cases a spatial intermingling of tumor and lymphocytic infiltrates, underpinned by robust activation of interferon (IFN) signaling pathways. Conversely, non-responsive or progressive (pNR) lesions were typified by heightened angiogenic signaling and oxidative metabolism, likely ensuring the requisite energy provision to facilitate the proliferation and architectural reconfiguration necessary for tumor progression. ST offers multidimensional insights into the complexity of TNBC and enables the prognostication of tumor behavior with precision. Collectively, these findings substantiate the utility of ST in affording novel perspectives for the refinement and personalization of therapeutic strategies.

ST is instrumental in crafting therapeutic strategies that are precisely targeted to distinct TME and specific cellular subpopulations, thereby optimizing therapeutic outcomes and curtailing superfluous adverse effects. Trastuzumab serves as an efficacious therapeutic for HER2-positive breast cancer; however, the development of resistance within a year is a common clinical challenge. A novel bispecific antibody, IMM2902, directed against CD47 and HER2, has been engineered to address trastuzumab-resistant breast cancer. Utilizing ST analysis in conjunction with multiplex immunofluorescence (mIFC) and *in vitro* assays, it was determined that IMM2902 is capable of robustly inducing macrophages to secrete C-X-C motif chemokine ligands 9 and 10 (CXCL9 and CXCL10), which are pivotal for the recruitment of T lymphocytes and NK cells to the TME. The integration of IMM2902 into the current therapeutic regimens holds the potential to markedly alter the clinical management of HER2-positive breast cancer, offering a novel avenue of hope for patients, particularly those with limited therapeutic options due to acquired resistance to existing treatments (46). An additional study, employing both single-cell and whole-tissue analytical approaches, has demonstrated a correlation between high levels of inner mitochondrial membrane protein (IMMT) and the immunosuppressive tumor immune microenvironment (TIME). This research substantiates the role of IMMT in the immunosuppressive phenotype of TIME, the proliferation of cancer cells, and mitochondrial adaptive mechanisms, thereby nominating pyridostatin as a promising candidate for targeted therapeutic development in precision medicine (47).

## 5 ST and prognosis assessment in breast cancer

ST analysis is a cutting-edge technique that plays a pivotal role in discerning cell subpopulations and molecular signatures linked to patient prognostication. This technology affords novel biomarkers that are instrumental for the categorical stratification of breast cancer, the prognostication of therapeutic responses, and the tailoring of individualized treatment paradigms. ST sequencing of disparate regions within clinical breast cancer tissue specimens has unveiled a higher prevalence of follicular helper T cells, quiescent dendritic cells, and plasmacytes within regions abundant in tumor cells, in contrast to areas rich in immune cells where there is a diminished presence of resting CD4^+^ memory T cells and T regulatory cells. The investigation has pinpointed activated leukocyte cell adhesion molecule (ALCAM), ADP-ribosylation factor-like protein 6-interacting protein 1 (ARL6IP1), and cyclin G2 (CCNG2) as potential immunoprotective agents in breast cancer pathology, while antizyme Inhibitor 1 (AZIN1), myoferlin (MYOF), and transforming acidic coiled-coil containing protein 2 (TACC2) are implicated as potential oncogenes. In locales of elevated tumor cell density, surfeit locus protein 4 (SURF4) and the lipid metabolic gene lysophospholipase I (LYPLA1) have been corroborated as biomarkers inversely related to favorable outcomes. Additionally, diacylglycerol o-acyltransferase 1 (DGAT1), LYPLA1, polymerase (RNA) II (DNA-directed) polypeptide K (POLR2K), and recombinant sphingomyelin phosphodiesterase 4 (SMPD4) are identified as influential factors that modulate patient survival outcomes (48). In the realm of TNBC, which lacks established biomarkers for outcome prediction, spatial profiling has uncovered that caspase 3 and cleaved poly (ADP-ribose) polymerase (cPARP), both indicators of cellular demise, are associated with inferior overall survival when in interaction with the epidermal growth factor receptor (EGFR). The absence of interplay between cells manifesting myoepithelial markers such as smooth muscle actin (SMA) and those indicative of cell cycle progression (mitotic figures) marked by phosphorylated histone H3 (pHH3) is correlated with diminished overall survival. Conversely, the interaction between stromal cells positive for vimentin and those exhibiting active receptor tyrosine kinase (RTK) signaling is associated with enhanced overall survival. These findings underscore the complexity and heterogeneity of the tumor microenvironment and highlight the potential of ST analysis in uncovering new therapeutic targets and prognostic biomarkers in breast cancer (49).

## 6 Potential challenges and future prospects for the clinical application of ST

These investigations underscore the transformative impact of ST on our apprehension of tumor heterogeneity, offering novel insights and methodological approaches for the molecular profiling of breast cancer, delineation of the TME, assessment of treatment responsiveness, and prognostic stratification, thereby holding the potential to catalyze the evolution of precision oncology in breast cancer. As technological advancements persist and applications are further explored, the anticipation is ripe for a cascade of transformative discoveries that are poised to innovate the diagnostic and therapeutic paradigms in breast cancer management.

Despite the progress made by ST profiling in discovering and identifying disease-specific and spatially specific factors, ST also has its limitations (**Table 1**). The spatial distribution within ST is subject to the positional integrity of tissue sections relative to the three-dimensional architecture of the organ or tissue and the fluctuating phases of disease progression. Given the considerable variability that can exist between tissue sections of different depths and orientations, there exists a legitimate query as to whether the spatial distribution captured by ST is comprehensively representative of the complete landscape and spectrum of variations within the multi-dimensional organ milieu (50, 51). The precision of pathological identification and selection is equally pivotal to the fidelity and congruence of ST maps and the inferred intercellular dynamics (52). The corpus of human specimens is constrained by the complexities surrounding sample acquisition, preservation, and transit, underscoring an urgent requirement for systematic, rigorously architected clinical research to elucidate the pathophysiological nuances of the disease (53). The translational journey of ST maps into clinical relevance is replete with challenges, stemming from discrepancies in molecular profiling and phenotypic manifestations between animal models and human subjects, as well as among various disease models (51). To encapsulate, the assembly and cartography of spatial profiles necessitate enhanced standardization and automation protocols. The clinical utility of ST is inextricably linked to the precision, reproducibility, and consistency of ST profiling, contingent upon the intricacies, severity, staging, pathological fidelity, and morphological exactitude of the disease context (54).

**Table 1:** Advantages, limitations, and application potential of spatial transcriptomics.

Feature	Description
AdvantagesLimitationsApplication Potential	1. Cellular Spatial Localization: Retains spatial information of cells, providing gene expression characteristics *in situ* (10). 2. *In Situ* Tissue Research: Advances the study of genuine gene expression of cells in tissue sites (10). 3. Broad Application Potential: Demonstrates potential in various fields such as tumor, embryonic development, and pathology (51). 4. Biological Interactions: Clarifies interactions between cells and the influence of the microenvironment (11). 1. ST Distribution Positional Influence: **The representativeness of ST distribution is influenced by the position of tissue sections and differences in disease stages** (50, 51). 2. Pathological Identification Accuracy**: The accuracy of pathological identification is crucial for the reliability of ST atlases** (52). 3. Human Sample Availability Limitation: **The limited availability of human samples restricts the conclusiveness of disease research** (53). 4. Animal Model-Human Disease Discrepancy: **Differences between animal models and human diseases pose challenges for the clinical application of ST atlases** (51). 5. Cost Issue: Higher cost compared to traditional RNA sequencing methods (54). 1. Revealing Cellular Heterogeneity: Identifying and locating different cell populations within tissues (10). 2. Drug Development: Identifying new biomarkers and drug targets (26, 27). 3. Spatiotemporal Dynamics Analysis: Revealing spatiotemporal dynamics within tissues (11). 4. Multi-Omics Integration: Combining with other omics data to provide a comprehensive perspective (57, 58).

With the rapid development of new ST technology, data acquisition is continuously improving, and challenges in ST resolution, sensitivity, throughput, and accessibility are being overcome (55). ST is compatible with paraffin-embedded tissues, providing the possibility for retrospective analysis of samples collected in biobanks. This will potentially allow for the systematic detection of various tissues and the reconstruction of the three-dimensional spatial structure of gene expression in organisms (51).

ST has emerged as a transformative approach in breast cancer research, offering innovative vistas and analytical tools that have propelled our comprehension of the TME and the intricacies of tumor heterogeneity. It has also been instrumental in fostering substantial advancements in the realm of breast cancer immunotherapy.

## 7 Conclusions

The present review synthesizes the burgeoning role of spatial transcriptomics in elucidating the intricate landscapes of breast cancer. It underscores the technology’s capacity to delineate the TME and identify cell subpopulations with unprecedented clarity. Despite its promise, spatial transcriptomics is still nascent and confronts hurdles such as cost-efficiency, data intricacy, and the need for analytical standardization.

Ongoing research must refine spatial transcriptomics to augment its resolution and scalability, while concurrently advancing bioinformatics methodologies to adeptly manage and interpret the voluminous datasets. Interdisciplinary cooperation, space multiple omics technology combines genomics, transcriptomics, proteomics and metabolomics, provides tissue space system in all or most of the gene expression level, has been used in neuroscience, development, cancer, plant biology and other fields, improve the biological insights on disease pathogenesis (56–58).

The burgeoning potential of spatial transcriptomics in breast cancer research is palpable, with the anticipation that it will engender transformative diagnostics and therapeutics. As the field matures, we anticipate its pivotal role in the realm of precision medicine, significantly impacting patient prognostication and treatment paradigms.
